# Hyper-fidelity depletion with discrete motion for pebble bed reactors

**DOI:** 10.1038/s41598-023-39186-3

**Published:** 2023-08-05

**Authors:** Yves Robert, Tatiana Siaraferas, Massimiliano Fratoni

**Affiliations:** University of California, Berkeley, Berkeley, CA 94709 USA

**Keywords:** Energy science and technology, Engineering, Mathematics and computing

## Abstract

Hyper-fidelity (HxF) depletion of pebble bed reactors (PBRs) is the capability to model depletion for every pebble while accounting for motion through the core. Previous HxF work demonstrated feasibility to deplete hundreds of thousands of stationary pebbles concurrently within reasonable timeframes. This work illustrates the second step towards HxF, coupling depletion with a discrete motion scheme. The model assumes an ordered bed with pebbles occupying fixed positions. Motion is simplified as discrete since pebbles move in straight lines from one set position to another. The methodology was implemented in Serpent 2, combined with its transport and depletion capabilities. Ad-hoc routines were developed ensuring compatibility with domain decomposition and pebble recirculation after each pass based on discharge criteria and fresh pebble insertion. Capabilities of HxF with discrete motion are demonstrated using a full-scale high-temperature gas-cooled reactor model. Specifically, an approach to equilibrium is performed, and example results are shown for in-core and discarded pebbles. The data illustrates how HxF provides unique insights into PBR fuel, producing information on statistical distributions rather than average values only, as obtained by traditional methods that rely on spectral zoning for depletion. Knowledge of these distributions can greatly improve analysis and assessment of PBRs.

## Introduction

Hyper-fidelity (HxF) depletion of pebble bed reactors (PBRs) is defined as the capability to model depletion for every individual pebble while also accounting for its motion through the core. This represents a shift of paradigm in solving the challenges associated with pebble bed depletion. A lengthy description of these challenges and the way they have been handled before is provided in an earlier paper demonstrating the computational feasibility of HxF^1,2^. For completeness, a short summary is provided here. Since the size of a pebble is small compared to the long neutron diffusion length in a graphite moderator, the neutron spectrum in each pebble is not self-determined, but rather it strongly depends on the content of the adjacent pebbles. Due to the continuous pebble recirculation and refueling, the fuel content of adjacent pebbles can drastically differ as their burnups are very different and not known a priori. A simple iterative process is not viable as a typical PBR core contains a few hundred thousand pebbles; therefore, past tools have addressed this challenge by dividing the core into macro zones (each containing tens of thousands of pebbles), within which a uniform fuel composition, thus neutron spectrum, is assumed^3–6^. These approaches are only capable of providing the average pebble behavior and lack verification for the simplifications they introduce. HxF, instead, resolves each pebble independently, which means it can provide detailed distributions of quantities of interests such as burnup, power, and temperature. As the limitations of a reactor system are often assessed based on the extremes rather than on average values (e.g., maximum power per fuel particle, maximum fuel temperature, etc.), the data generated through HxF are expected to greatly improve our capability to assess the safe operation of PBRs. Furthermore, this higher resolution method can serve as verification for the traditional spectral zone methods.

The ultimate goal of HxF is to integrate discrete element modeling (DEM) for realistic pebble motion, Monte Carlo neutron transport for power distribution and fuel burnup calculations for each pebble, and a thermal–hydraulic model to determine temperature distribution. In order to meet this ambitious goal, rather than implementing all parts in a single attempt, a progressive approach was adopted. The first step was to demonstrate the feasibility of depleting a large number of materials in a reasonable timeframe without relying on supercomputing. It was proven^1,2^ that it is possible to deplete up to 0.5 million pebbles concurrently using relatively limited computational resources and within a timeframe of five to ten days. More significantly, it was demonstrated that HxF is a powerful tool to enhance our understanding of PBRs by revealing valuable insights on the fuel and reactor behavior, such as power peaks and burnup distribution at discharge, otherwise not available using traditional tools. Finally, even if HxF is not suitable for rapid scoping analysis, it provides a verification instrument for lower-fidelity tools.

That first step focused on proving the concept of HxF and assumed no pebble motion. This manuscript, instead, documents the second step towards the development of HxF, where depletion is coupled to pebble motion. The initial implementation does not use, yet, DEM, but a simplified model for pebble motion referred to as discrete motion. Such model assumes an ordered bed with fixed pebble positions within which pebbles move in straight lines from one set position to another. This paper presents (Section “Methodology”) the theory and implementation of HxF with discrete motion using the Monte Carlo code Serpent 2^18^ that, based on previous work, possesses unique enabling features for HxF. The developed methodology is demonstrated (Section “Test case”) by modeling a large-scale high-temperature gas-cooled reactor (HTGR), and the results are illustrated and discussed (Section “Results”).

## Methodology

### Discrete motion approach

Discrete element models (DEMs) have been applied to PBRs to determine the trajectory of each pebble during its lifetime^7–13^. For example, OpenFOAM and its DEM solver particleFoam have been used for simple applications in PBRs^8^. In addition, Serpent embeds an internal coupling with OpenFOAM allowing for reading OpenFOAM-formatted files and changing information (e.g., temperature, density) on-the-fly. However, two limitations make this approach challenging. First, although the communication interface between OpenFOAM and Serpent can be leveraged, changing the pebble bed configuration cannot be done in the current state of the latter code. The cartesian search mesh, necessary for Serpent to explicitly model pebbles, needs to be updated when the position of the pebbles is changed. However, the search mesh is static in Serpent 2, and modifying this behavior would require extensive development efforts and profound modifications of the source code structure. In addition, it would be necessary to exit the code at every motion step, modify the input, and rerun Serpent. This process results in significant computational time for re-creating the geometry and materials and importing the saved compositions, in addition to increasing the complexity of the procedure. Second, although the OpenFOAM DEM solver has been used in the past for simulating the core loading^14^, it has not been used with pebble recirculation. Overall, the implementation of this solver is not advanced enough and does not include the necessary features to make a full-scale pebble bed recirculate for multiple passes.

**Figure 1:**
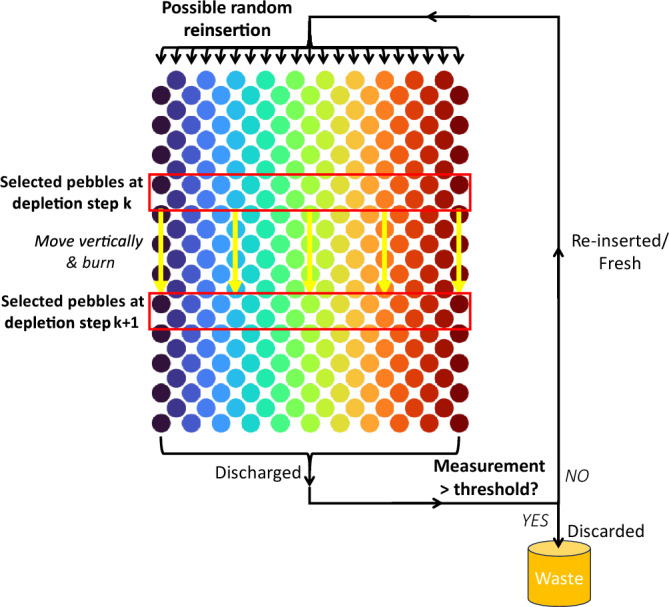
Schematic representation of the discrete motion approach (downward motion case). Colors represent possible trajectories and domains used for the simulation.

For these reasons, the initial coupling of pebble motion and depletion is implemented, assuming a simplified motion model that leaves the search mesh static by keeping the pebble positions unchanged throughout the simulation (Fig. 1). The motion is then represented by moving pebbles' compositions from one position to another. A routine was added to the Serpent 2 source code that moves compositions and accounts for discarding depleted pebbles and inserting fresh ones. It is assumed that pebbles move in straight lines, upward or downward, according to the type of PBR being represented.

### Implementation

The discrete motion feature in Serpent has two main components: the composition shuffling and the pebbles' recirculation handling. Changing the domain of pebbles was also examined. For this implementation, some simplifications needed to be done as explained in the Section “Simplifications”.

**Figure 2:**
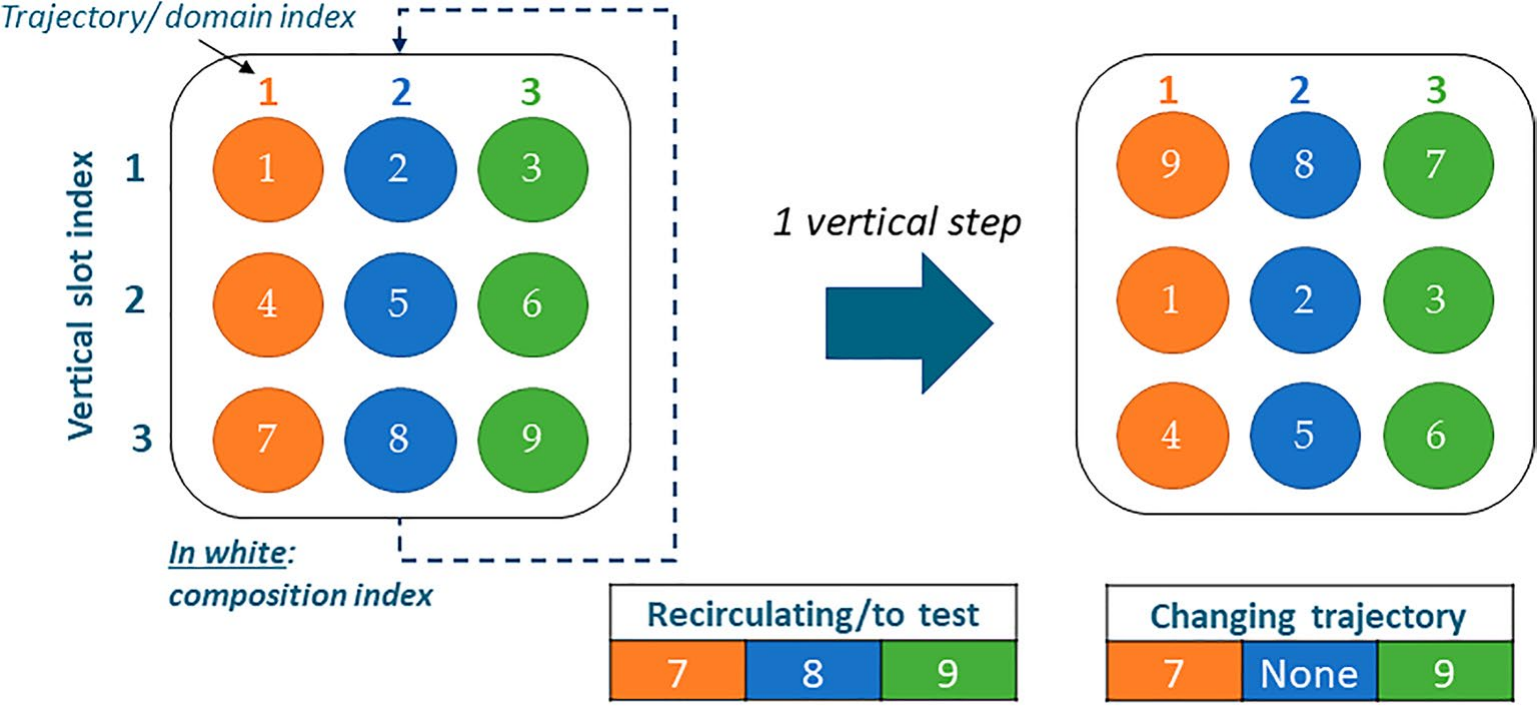
Example of discrete motion for a two-dimensional PBR. Composition IDs are shown in white. The pebble positions are fixed, and compositions move downward in a discrete way. Bottom pebbles recirculate and are tested for burnup. Pebbles are reinserted in a random position at the top—pebbles changing trajectory change domain and their data need to be communicated between the domains.

A simplified illustrative example of a discrete motion step with a two-dimensional PBR and nine compositions is shown in Fig. 2 (the direction of pebbles is only an example and can be changed as needed). In this example, pebbles 1 to 6 move down by one vertical slot after one step. Pebbles 7, 8, and 9 are tested for burnup: 7 and 9 are reinserted at the other extremity in a randomly selected trajectory; 8 is discarded and its composition is replaced with fresh fuel concentrations, while keeping the same index to reduce the number of materials to initialize in Serpent.

#### Compositions shuffling

The capabilities of reading and modifying compositions in Serpent 2 were implemented as follows. When using the automized division of materials that Serpent applies to create the pebbles from the input pebble bed file, the parent fuel material is divided into identifiable zones, each having a specific ID number corresponding to a specific composition. These composition IDs are sorted in the same order as how the positions are defined in the explicit stochastic geometry and are stored in the ID vector ${I}^{\left(0\right)}$. The routine was modified so that at any step $k>0$, Serpent reads the current ID order ${I}^{\left(k-1\right)}$ in which compositions are and the new ID order ${I}^{\left(k\right)}$ in which compositions should be, and calculates a transition operator ${T}^{\left(k\right)}$ with the following equation:

1 $${I}^{\left(k\right)}={T}^{\left(k\right)}{I}^{\left(k-1\right)}$$

Then, a new list of fuel materials is created from the current one, based on the transition operator. This method can be applied to any reactor or geometry as long as the zone numbering is managed correctly. For this application, compositions are vertically shuffled at each step, replicating an upward or downward motion based on the type of PBR.

#### Pebbles recirculation handling

Most PBRs are envisioned to apply a multi-pass fuel management scheme, meaning when a pebble reaches one end of the core, it must be discharged, tested for burnup, if needed replaced by fresh fuel, and reinserted at the other end. Therefore, it is necessary to account for pebble recirculation to represent this operation accurately. Discrete motion reproduces such behavior by assigning the composition of a recirculating (or a fresh fuel composition) to a pebble ID located at the other extremity of the core. The position of the pebble can be pre-determined or, as more common, be randomly chosen.

The newly developed capability for pebble recirculation tracks the number of passes and tests pebbles against a preset discarding criterion. Options for this criterion include a maximum burnup value, a maximum quantity of a set isotope (e.g., ^137^Cs), or a maximum number of passes the pebble can go through the core (these different options are not all realistic but rather include criteria that have been used in other tools and benchmarks). The composition of discarded pebbles is stored in a separate data file for used-fuel characterization.

#### Pebbles changing domain

One key enabling feature for HxF is the domain decomposition method that Serpent provides for burnup. In order to reduce memory requirements, Serpent splits the volumes to burn into multiple zones (domains) and distributes them among the computing nodes. Domains are continuous (cuboids, cylinders, or wedges), and materials information is not shared across domains. In practice, this method results in a division of the memory requirement, largely dominated by materials data, by the number of domains used. Still, when discrete motion is applied, it occurs that materials move from one domain into another. To overcome this issue, data of materials that change domain are stored in an external file that is then read to populate the target domain. This process increases computing time by roughly 40% due to the communication between domains during transport when a neutron coming from a domain interacts with a material in another domain and data processing. Nevertheless, the benefits of domain decomposition in terms of drastic memory reduction greatly overshadow the additional time.

#### Simplifications

At this stage, the methodology illustrated above includes some simplifications. In addition to limiting pebbles to only occupy set positions, it is typically assumed that pebbles move as vertical channels without cross-mixing^3,4,15^. Furthermore, changes in the core geometry, such as the conic regions typically found in PBRs, are not considered. These limitations are not intrinsic to the discrete motion model but would require significant changes to the method; therefore, it was decided to address them in future work employing DEM. Although it is possible to maintain a more realistic pebbles distribution in the core, they are typically arranged in a regular lattice. This simplifies the generation of motion sequences without significantly affecting the expected results due to the large neutron diffusion length in the system.

## Test case

**Figure 3:**
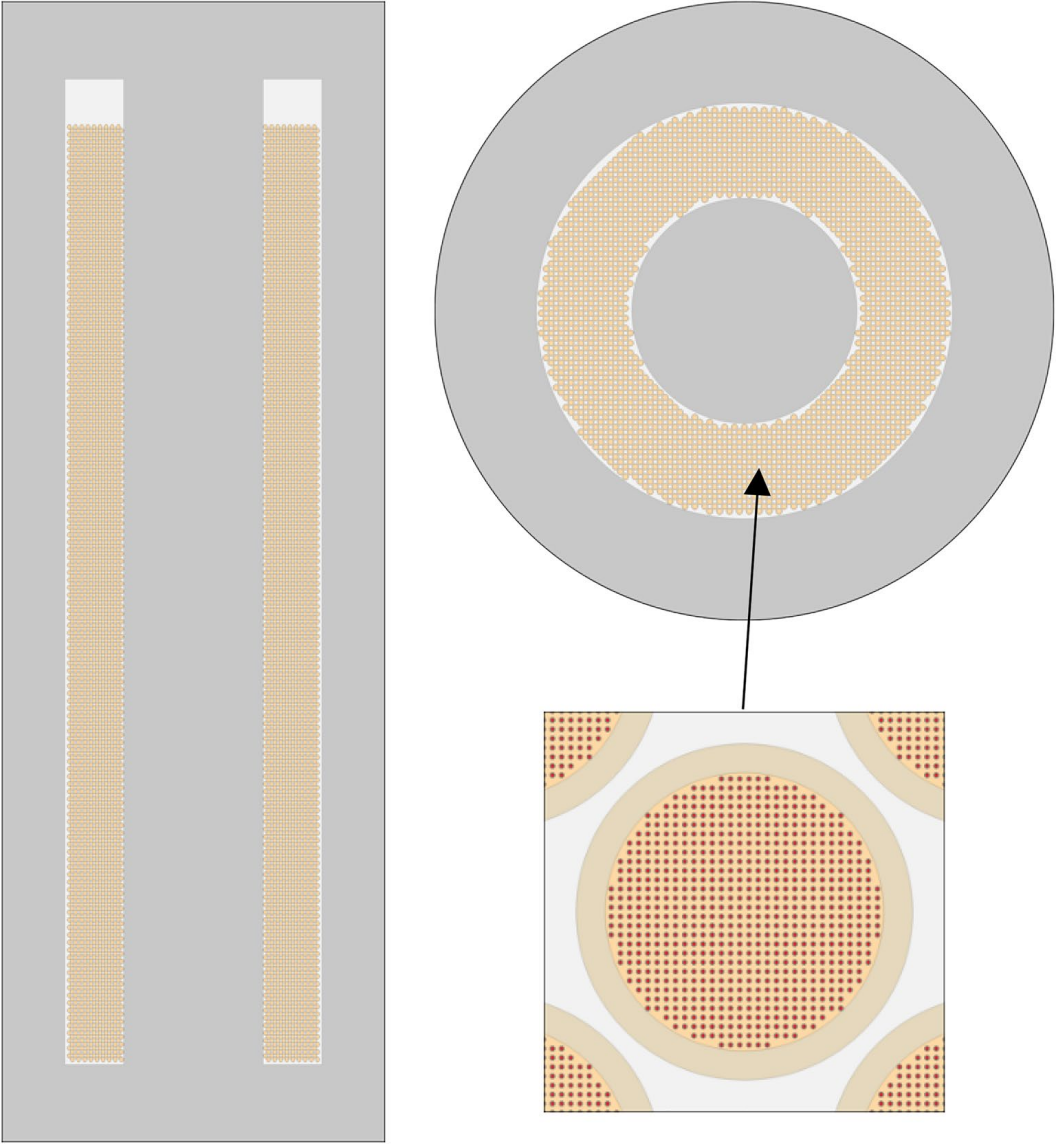
Test case model vertical (left) and horizontal (top right) cross section and pebble model (bottom right).

The capabilities of HxF with discrete motion are demonstrated by determining the equilibrium composition for a full-scale HTGR core. The model, depicted in Fig. 3, incorporates typical geometry, dimensions, and material compositions based on the PBMR–400 design. Cycles and thermal–hydraulic parameters are arbitrarily assumed. Table 1 provides dimensions, materials, and other data. Although certain parameters are sourced from design documentation^16^, it is important to note that this study serves as a demonstration of HxF capabilities and does not aim to establish a benchmark solution. To match the assumptions made for discrete motion, the geometry is simplified with a fully cylindrical active region surrounded by 90 cm-thick axial and radial reflectors and a 100 cm-radius inner graphite reflector. The resulting 1100 cm-high, 85 cm-wide pebble bed contains 451,360 pebbles. At the top of the bed is a 50 cm-high He-filled space. Pebbles are distributed in a face-centered cubic (FCC) lattice. The fuel is in the form of 9.8 wt%-enriched UO_2_ kernels contained in a simple cubic lattice of 15,000 TRISO particles without clipping. The fuel temperature is set at 1200 K, whereas the rest of the materials are assumed to have a uniform temperature of 900 K. Although the methodology can use a velocity profile, for simplicity, a flat profile is assumed, meaning that pebbles belonging to the same row move with the same velocity.

**Table 1:** Test case parameters^16^.

Component		Parameter	Value
Core		Total power	400 MWth
		Active height	1100 cm
		Total core height	1150 cm
		Inner reflector radius	100 cm
		Active radius	185 cm
		Outer reflector thickness	90 cm
		Non-fuel temperature	900 K
Pebbles	Bed	Number of pebbles	451,360
		Packing fraction	61.0%
		Layout	FCC
		Pitch	3.115 cm
		Uniform pebbles velocity	10.87 cm/day
		Discharge rate	4514 pebbles/day
	Matrix/Shell	Graphite density	1.704 g/cm^3^
		Graphite shell density	1.750 g/cm^3^
		Pebble outer radius	3.0 cm
TRISO particles	Lattice	Layout	SC
		Number of TRISO	15,000
		Packing fraction	9.1%
		Pitch	0.0804 cm
	Fuel	Outer radius	0.02 cm
		Form	UO_2_
		Enrichment	9.8 wt%
		Temperature	1200 K
		Density	10.4 g/cm^3^
	Buffer	Outer radius	0.03 cm
		Density	1.050 g/cm^3^
	IPyC	Outer radius	0.0335 cm
		Density	1.900 g/cm^3^
	SiC	Outer radius	0.0370 cm
		Density	3.180 g/cm^3^
	OPyC	Outer radius	0.0405 cm
		Density	1.900 g/cm^3^

### Motion sequence

As aforementioned, the shuffling of the compositions is based on a pre-determined series of ID lists reproducing the motion of the pebbles. Since the bed is arranged in an FCC lattice, the motion sequence is relatively simple. The bed at step $k$ is represented as a matrix ${M}^{\left(k\right)}$ in which each row corresponds to axially aligned pebbles, and each column corresponds to radially aligned pebbles. With this representation, the matrix element ${m}_{i,j}^{\left(k\right)}$ corresponds to the composition at the slot of respective row and column indices $i$ and $j$, at step $k$. Every two consecutive rows in the FCC lattice are labeled with the same index. The matrix is sorted in ascending index order, and a downward shift of compositions is applied from one step to the next. The magnitude $n$ of the vertical shift (i.e., the number of rows by which compositions are shifted) depends on the selected size for the burnup step. Therefore, if the overall matrix of the bed is of size $\left({N}_{r}, {N}_{c}\right)$, the top ${N}_{r}-n$ rows are shifted downward, and the bottom $n$ rows are moved to the top of the bed. Assuming that the first pebble out is the first pebble in and radial insertion is not controlled, the recirculated pebbles maintain the same stratification but are randomly reassigned to a column. The correlation between two consecutive steps can then be represented as follows (where *i* = 0 is the bottom row):

2 $${m}_{i,j}^{(k)}=\left\{\begin{array}{c}{ m}_{i+n,j}^{(k-1)} \forall i\in \left\{1, {N}_{r}-n\right\}, \forall j\in \left\{1,{N}_{c}\right\} , \forall k>0 \\ {m}_{i-{N}_{r}+n,j{\prime}}^{(k-1)} \forall i\in \left\{{N}_{r}-n+1, {N}_{r}\right\},\forall {j}{\prime}\in \left\{1,{N}_{c}\right\} ,\forall j\in \left\{1,{N}_{c}\right\} , \forall k>0\end{array}\right.$$

The new state ${M}^{\left(k\right)}$ is then converted into the ${I}^{(k)}$ vector and written to a step-dependent file which the Serpent discrete motion routine uses:

3 $${I}^{(k)}=\mathrm{vec}\left({M}^{\left(k\right)}\right), \forall k$$

Additionally, the slots which contain recirculating compositions (i.e., of the $n$ last rows) are stored in a recirculation file for assessment against the discharge criterion. It is to be noted that the motion sequence does not perform discharge and refueling. The Serpent 2 shuffling routine entirely handles this process. The motion sequence can be changed based on the specific geometry and flow direction, and it uses indices generated with any external method, regardless of complexity.

The test described here assumes ${N}_{r}$=124 rows, and ${N}_{c}$=3640 columns (or trajectories). An initial motion step of $n$=61 is set to accelerate convergency towards the equilibrium core. Then, finer motion steps of $n$=11 are applied. Such step corresponds to a shift of 96.5 cm. That means 8.9% of the bed is recirculated at each step, and, given the pebble velocity, each burnup step lasts 8.9 effective full power days.

### Computational setup

Along with the described sequence and between each motion step, a transport and depletion calculation is run by Serpent to determine the neutron population distribution, interactions with core materials, and resulting changes in compositions. The ENDF/B-VII.0 nuclear data library^17^ is loaded once at the beginning of the simulation and stored throughout, obviating the need for reloading. Drawing from insights gained in previous research^1,2^ domain decomposition and pebble-wise automated burnable materials division are employed. These options facilitate the definition of a unique parent fresh fuel material, which is then efficiently subdivided into individual zones per material. This approach simplifies the input process, reduces simulation and memory requirements, and streamlines the overall computational workflow. Additionally, Serpent optimization mode 1 is applied, utilizing a non-unionized energy grid, and performing on-the-fly cross-section calculations through the direct tally approach. This choice further mitigates memory demands, enhancing computational efficiency. Starting from an initial random fuel composition, coarse steps are applied with 10^6^ inactive and 10^7^ active neutrons. Once a first equilibrium is obtained with this high uncertainty, large motion step sequence, a new simulation with 10^7^ inactive and 10^8^ active neutrons is run. Seventy full passes are simulated, each corresponding to 100 days, and the discarding criterion for pebbles is based on ^137^Cs concentration, whose threshold value is set at 2.2238 × 10^−4^ mol/pebble, which roughly corresponds to a desired threshold value of 92 MWd/kg_HM_. No predictor/corrector scheme is used in this study, instead a non-iterative depletion method is applied.

## Results

This section illustrates some of the results obtained using HxF with discrete motion for the HTGR test case. In particular, it discusses the convergency to equilibrium and analyzes the equilibrium parameters, both for in-core and discarded pebbles.

### Statistical considerations

#### Equilibrium state

**Figure 4:**
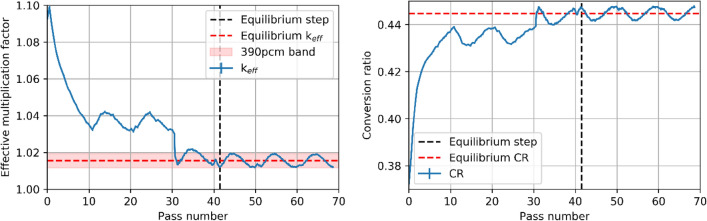
Evolution of global core parameters as a function of passes: multiplication factor (left) and CR (right).

Determining the convergence criteria is essential when searching for equilibrium in a PBR. Two different metrics were used in this work. First, the evolution of global parameters, such as the multiplication factor k_eff_ and the conversion ratio (CR), indicate the reactor's overall state. In this context, the equilibrium state is determined when these parameters have consistent trends with the fine motion step for three complete core cycles. Figure 4 shows how these two parameters have similar behaviors.

A first oscillatory trend is observed from 10 to about 30 passes. Oscillations result from motion steps substantially larger than the diffusion length of neutrons in the core. This first simulation stage with large burnup is beneficial to decrease the computing time to reach equilibrium. Then, a drastic reduction in the burnup step was applied, resulting in smaller motion steps. As soon as this step size reduction happened, the multiplication factor dropped by around 1500 pcm, and the CR increased by 7 × 10^−3^. This trend is explained by the lower number of fresh pebbles inserted into the core. Although some oscillatory behavior remains, the core is considered in an equilibrium state when the multiplication factor stays within a band of ± 390 pcm, that is, after 42 passes. Oscillations are most likely caused by the dynamic nature of PBRs operation with pebbles motion. However, they can be influenced by the statistical uncertainty of the transport processes and the discrete nature of depletion calculations with batches of fresh pebbles inserted and used pebbles discarded. Every simulated step after this is regarded as an equilibrium state with a different configuration. In the results presented below, equilibrium average values refer to the average of a given quantity over 297 states (corresponding to 27 passes), whereas representative values for a single state refer to the last equilibrium state simulated. The average equilibrium multiplication factor and CR are 1.01554 ± 18 pcm and 0.44472 ± 14 pcm, respectively.

**Figure 5:**
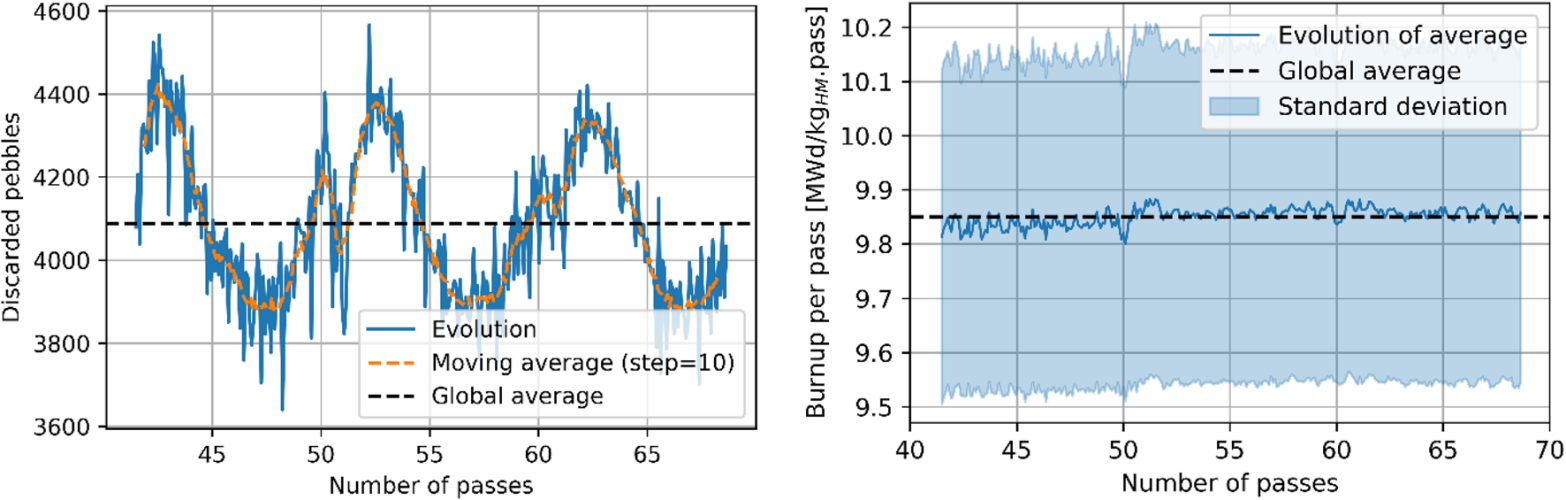
Evolution of global discarded pebbles parameters as a function of passes: number of discarded pebbles (left) and average discarded pebbles burnup, normalized by the number of passes (right).

Further evidence of achieved equilibrium is sought by analyzing discarded pebbles. Figure 5 shows the evolution of the number of discarded pebbles (thus, of the inserted fresh pebbles) as a function of the total number of passes simulated. The value oscillates around 4088 pebbles per step, between 3640 and 4566, corresponding to about 410 to 515 pebbles/day. Once again, these variations are interpreted as small enough to assume an equilibrium state. The increase of the discarded pebbles at around 50 passes matches the one of the multiplication factor previously observed. Figure 5 also shows the average burnup per pass and how this value, after reaching equilibrium, remains almost constant at around 9.85 MWd/kg_HM_.

Overall, it is to be observed that the criteria set to determine equilibrium are arbitrary. Given the stochastic nature of PBRs, a core at equilibrium will always present an oscillatory behavior; therefore, it will have to be the responsibility of the modelers to apply their best judgment in determining acceptable criteria for equilibrium.

In terms of computational requirements, after a 36.9-min initialization, the average transport process time for the initial depletion steps is 2.3 min, whereas for subsequent more accurate steps it increases to 19.6 min. The burnup and data processing times are relatively shorter, taking 1.2 and 1.1 min, respectively. The overall simulation time, conducted on 20 2.1 GHz Intel Xeon Gold 6230 nodes, amounts to 148 h. Each node is allocated 18.7 GB of memory, with the majority utilized for domain materials data (55.7%), cross sections (25.2%), and calculated and tallied results (9.0%). The remaining memory corresponds to miscellaneous data.

#### Uncertainties and peaking factors

Performing Monte Carlo calculations in large-scale models requires quantifying statistical uncertainties. First, the multiplication factor is not a limiting factor for the test case. The maximum statistical uncertainty obtained during fine steps is 22 pcm, which is small compared to the parameter variations. The main reason for simulating many neutron histories lies in the statistical uncertainty of pebble-wise detectors, such as the flux and power tallies. The highest values are found in pebble-wise power tallies due to the small size of the TRISO particles in which fissions are scored. The results are summarized in Table 2. Most pebbles (95%) have less than 6% uncertainty on the neutron flux and less than 12% on power.

**Table 2:** Summary of statistical uncertainties in pebble-wise detectors.

Detector	Average (%)	Standard Deviation (%)	Minimum (%)	Median (%)	75% Percentile (%)	95% Percentile (%)	Maximum (%)
Thermal flux (E < 1.86 eV)	2.3	0.9	1.0	2.0	2.7	4.3	6.7
Epithermal flux (1.86 eV < E < 0.1 MeV)	2.5	0.9	1.1	2.1	2.9	4.5	8.6
Fast flux (E > 0.1 MeV)	3.4	1.2	1.5	3.0	3.9	5.9	12.8
Power	7.4	2.1	3.1	6.7	8.3	12.0	22.9

**Figure 6:**
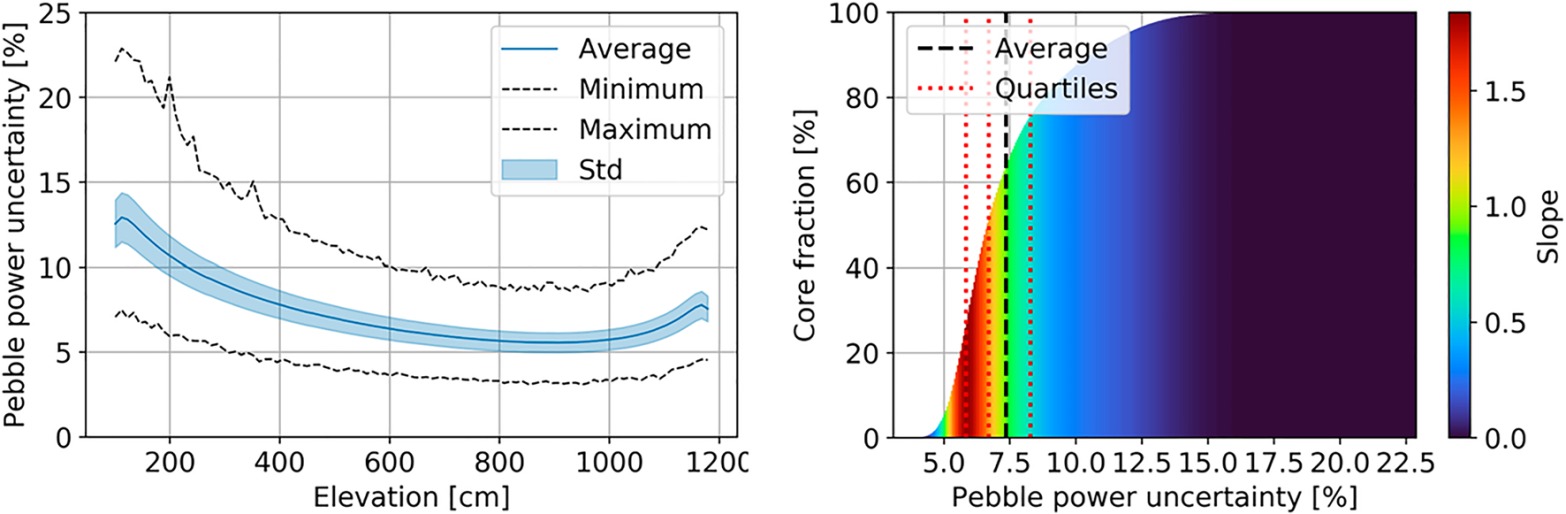
Axial profile (left) and cumulative statistical distribution (right) of the pebble-wise power statistical uncertainty.

Nevertheless, as Fig. 6 suggests, the highest uncertainties are, as one can expect, at the bottom of the core, where there is the lowest number of neutron/nuclide interactions. This zone corresponds to where the fuel is the most burned and ready to be discharged, which results in lower power production. In addition, as the histogram shows more clearly, the fraction of pebbles having a very high uncertainty in power and flux is small.

**Figure 7:**
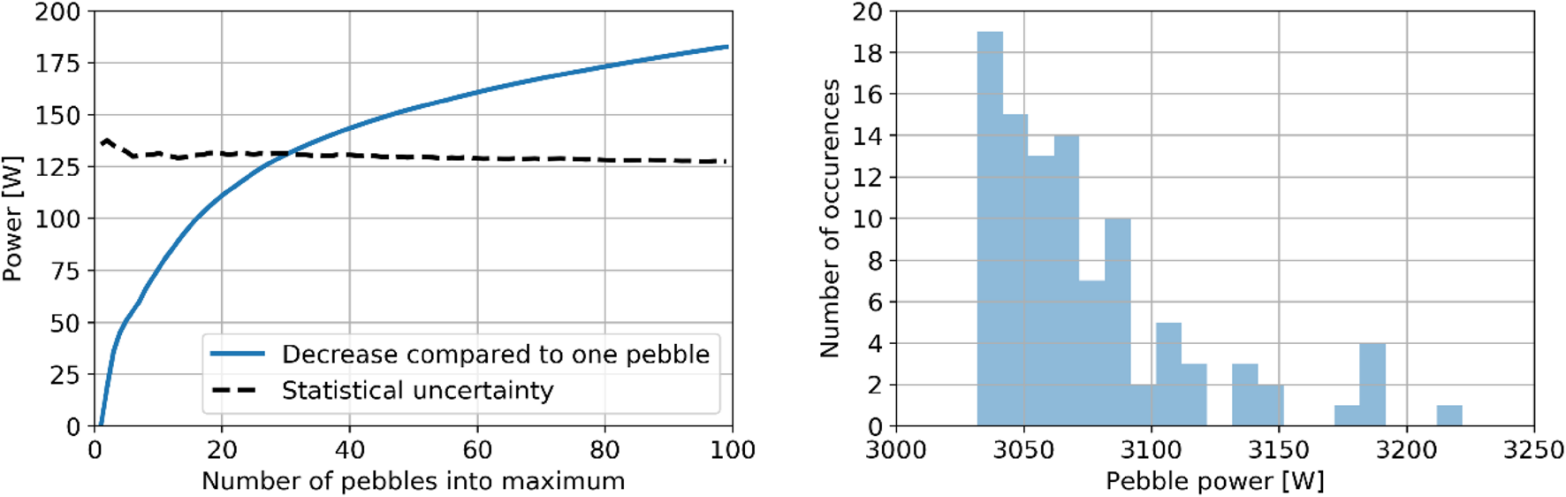
Evolution of the maximum power and its associated statistical uncertainty at equilibrium as a function of the number of values averaged to obtain this maximum power (left) and maximum 100 pebble-wise powers found at equilibrium (right).

The statistical uncertainty could, instead, have a more significant impact on extreme, maximum, and minimum values. For example, when calculating the pebble power peaking factor, it is impossible to establish to what extent the value for maximum power is a real outlier or a statistical artifact. Nevertheless, Fig. 7 shows that the pebble peaking factor only changes by roughly 5% when calculated using as peak the single highest power value and when using as peak the average 100 highest values. The same is true for neutron flux.

### In-core equilibrium data

The following sub-sections provide a summary of the distribution of key in-core parameters at equilibrium, such as neutron flux, burnup, and power*.* It is essential to understand that the data depend highly on the number of times pebbles went through the core. In fact, as the discard criterion is based on the content of ^137^Cs in the pebble, the number of passes varies depending on the individual history. Table 3 provides the count of pebbles in the core over multiple equilibrium representations grouped by pass number. The number of pebbles is almost evenly distributed between 1 and 9 passes, each accounting for around 10% of the core. Pebbles at the 10th pass, instead, make 8.2% of the total inventory, and an 11th pass is highly improbable. This suggests that pebbles are mostly discarded after 9 and 10 passes, and very few go through the core for 11 passes. Additional discussion on this matter is provided later on concerning discharge burnup.

**Table 3:** Average in-core pebble inventory over multiple equilibrium states.

Pass number	Average number of pebbles	Pebbles fraction [%]
1	46,152	10.2
2	46,206	10.2
3	46,264	10.2
4	46,224	10.2
5	46,076	10.2
6	45,892	10.2
7	45,802	10.1
8	45,811	10.1
9	45,889	10.2
10	37,031	8.2
11	14	3E−03
Total	451,360	100.0

#### Neutron flux

**Figure 8:**
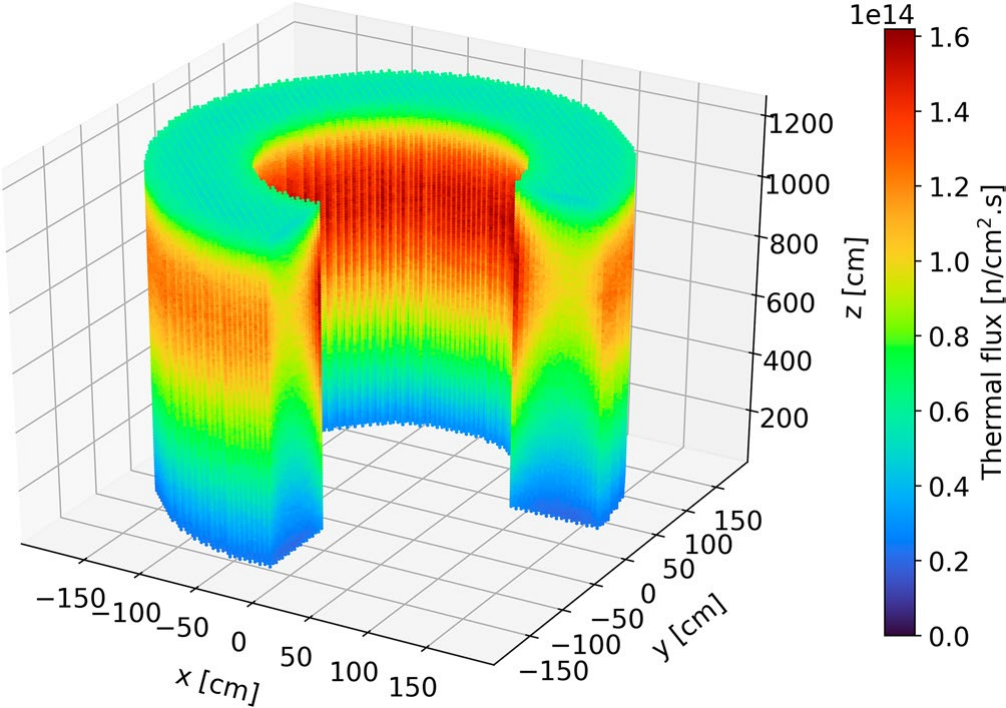
Thermal neutron (< 1.86 eV) flux in each pebble in the core at a representative equilibrium step.

**Figure 9:**
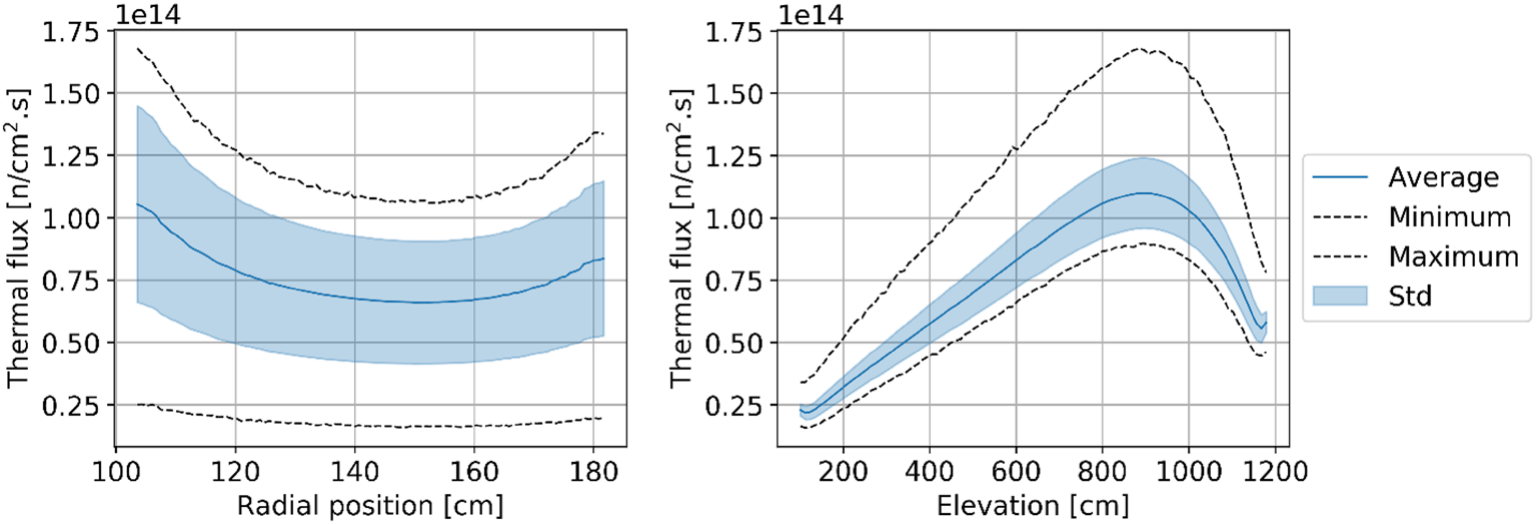
Radial (left) and axial (right) average thermal neutron (< 1.86 eV) flux profiles at equilibrium.

**Figure 10:**
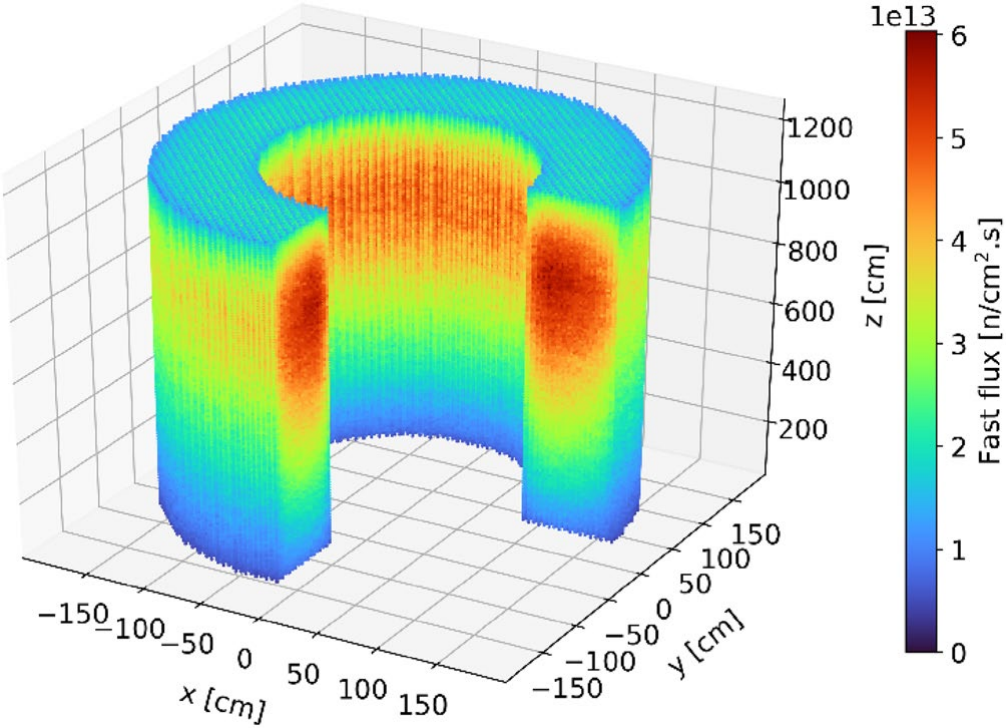
Fast neutron (> 0.1 MeV) flux in each pebble in the core at a representative equilibrium state.

**Figure 11:**
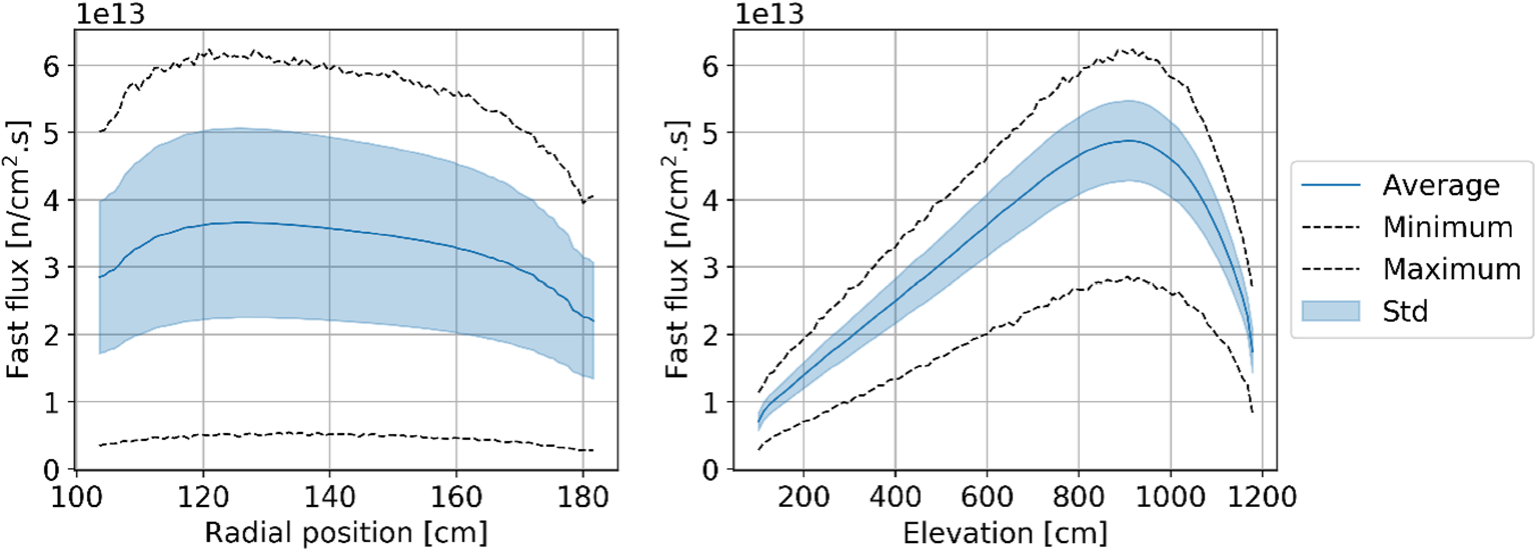
Radial (left) and axial (right) average fast neutron (> 0.1 MeV) flux profiles at equilibrium.

Figures 8, 9, 10, and 11 show the spatial distribution of thermal (E < 1.86 eV) and fast (E > 0.1 MeV) neutrons in the equilibrium core. As expected, the thermal flux peaks near the radial reflector and toward the top of the core. Indeed, neutrons are thermalized by the reflector, and once they re-enter the core, they do not travel long distances before being absorbed. In addition, the hollow-cylindrical nature of the core leads to a geometrical peak around the axial and radial centers of the bed while leading to neutrons leakage around the corners. However, since pebbles are inserted from the top and discharged at the bottom and due to the large accumulated burnup per pass, pebbles experience a more significant flux, both thermal and fast, towards the top of the core.

**Figure 12:**
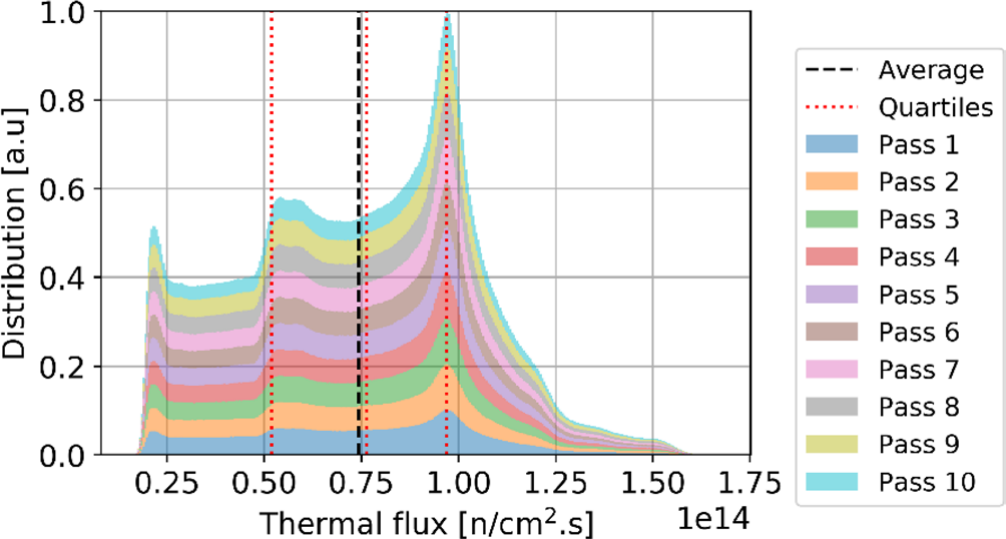
Cumulative thermal flux statistical distribution per pass over all equilibrium states, normalized over the maximum count (the envelope of the stack represents the global distribution).

**Figure 13:**
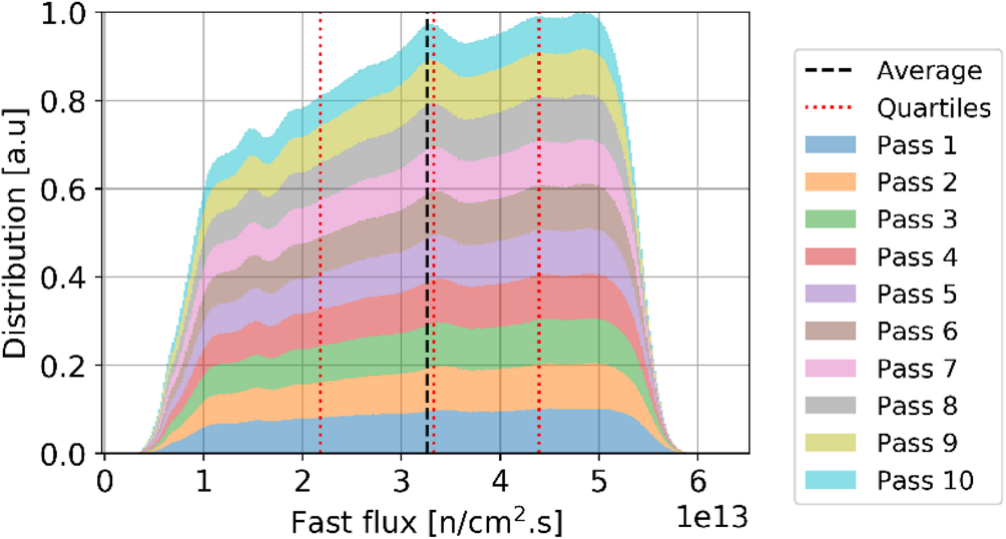
Cumulative fast flux statistical distribution per pass over all equilibrium states, normalized over the maximum count (the envelope of the stack represents the global distribution).

Two observations can be made regarding the statistical distribution of the thermal flux per pass in the core at equilibrium, shown in Fig. 12. Please note that in this plot and all other plots in this section showing per pass information, pass 11 does not appear because the sample size is too small to be visible. On the one hand, the thermal flux distribution is similar regardless of the pass number. On the other hand, the distribution shows clear flux peaks at around 0.2, 0.6, and 1.0 × 10^14^ n/cm^2^ s. Each of these peaks can be linked to identifiable core regions and is noticeable in Fig. 8. The low peak corresponds to the bottom region of the core, with the most burned fuel and thermal leakage; pebbles with the median peak value are found at the top of the core and directly above the low peak region; the highest peak (which also has the highest value) corresponds to the central region where pebbles are sufficiently far from the reflector. Finally, a few pebbles at the core's top inner and outer edges experience the highest values. The same distribution for fast neutrons (Fig. 13) shows that the maximum values slightly decrease with the number of passes.

#### Fuel utilization

**Figure 14:**
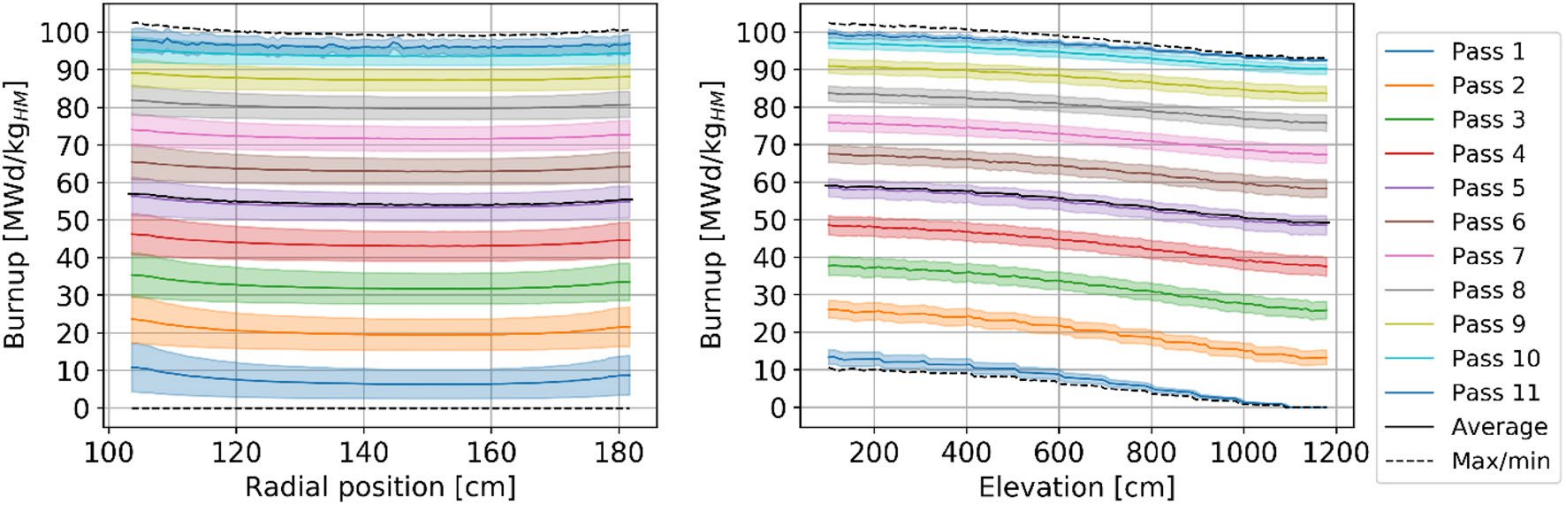
Radial (left) and axial (right) burnup profile per pass at equilibrium.

Figure 14 illustrates the spatial in-core distribution of burnup. The radial profile shows small peaks around the edges due to pebbles accumulating burnup more rapidly when closer to the reflector, particularly during the first four passes (Table 4). As pebbles are reinserted in a random radial location, the radial burnup profiles flatten with the number of passes. The axial burnup profile shows a monotonic increase behavior as pebbles descend through the core and accumulate burnup. The step-like behavior, noticeable mostly for pebbles in the first few passes, is artificial. It is caused by the discrete motion, moving a bit less than 1/11th of the core active height at each step. In any case, this artifact does not impact the trend and disappears as pebbles are randomly reinserted in different radial positions.

**Table 4:**
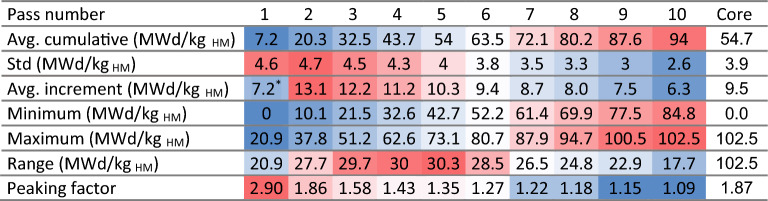
Burnup statistics as a function of the number of passes.

*The first pass average increment only shows the average burnup, whereas values for other passes correspond to the average burnup difference.

The colors are mapped from blue (lowest values) to red (highest values).

**Figure 15:**
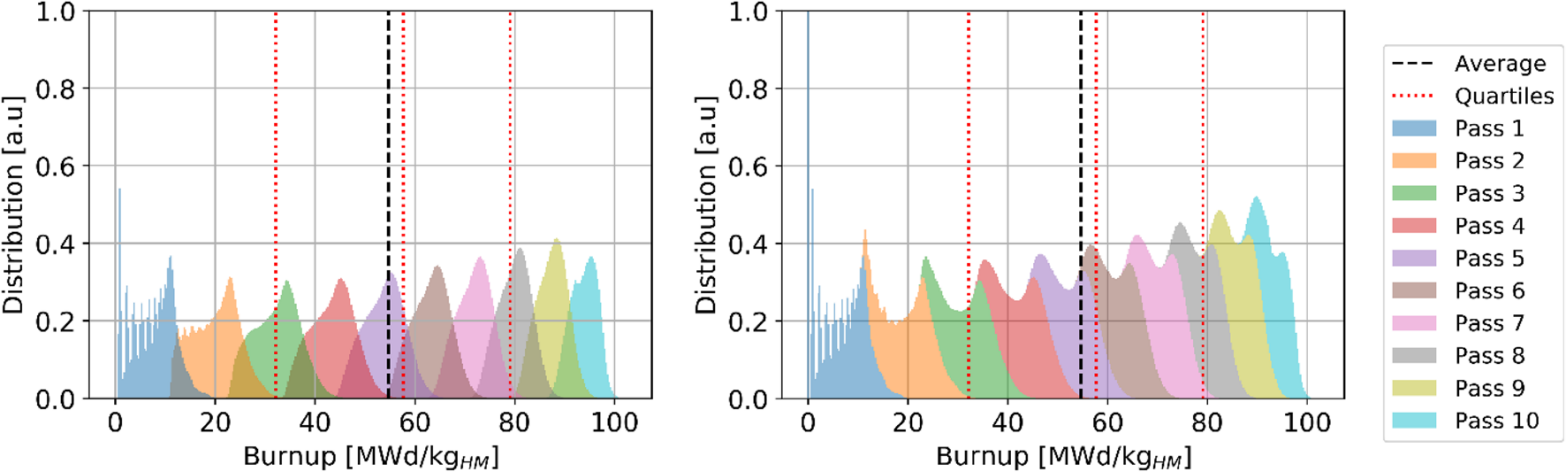
Burnup statistical distribution per pass over all equilibrium states, individual pass (left), and cumulative (right), normalized over the maximum count.

Figure 15 and Table 4 provide statistical data on burnup as a function of the number of passes. Two phenomena are worth noticing. First, the burnup distribution of pebbles during the first pass shows two anomalies: the large peak at zero burnup representing the fresh pebbles inserted in the core and the artificial multi-peak behavior due to the discrete motion approach. Second, the two peaks in the cumulative distribution at each pass resulting from the discrete nature (real in this case) of each pass through the core. In other words, pebbles with different numbers of passes have overlapping burnups, which, when cumulated, generates patterns that are hard to attribute to a pass number if one does not have access to pass-dependent data.

#### Power

**Figure 16:**
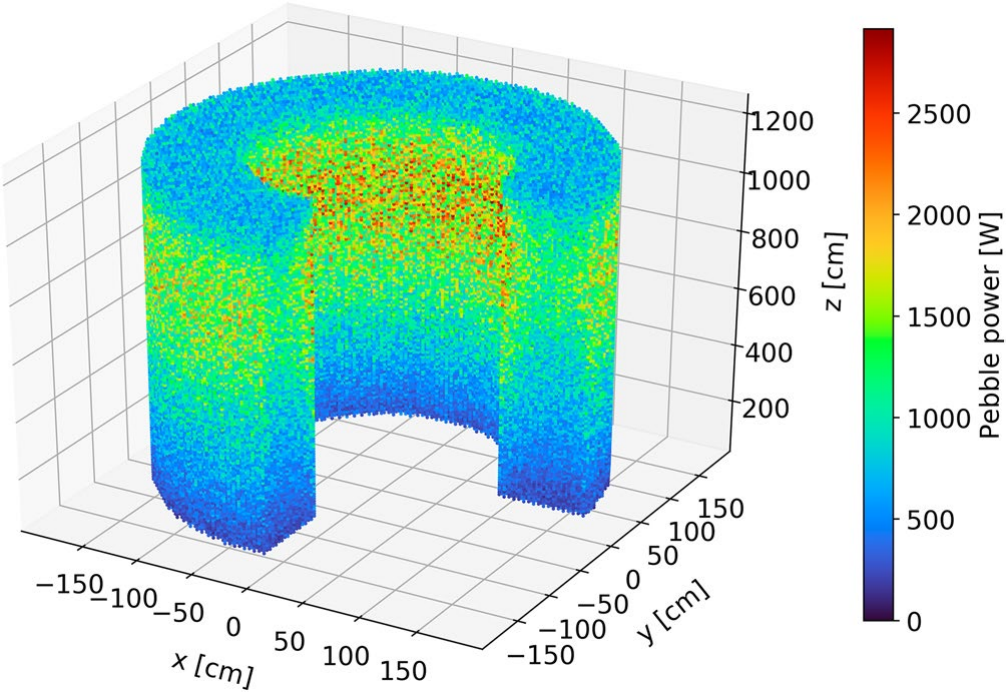
Power per pebble in the core at a representative equilibrium state.

**Figure 17:**
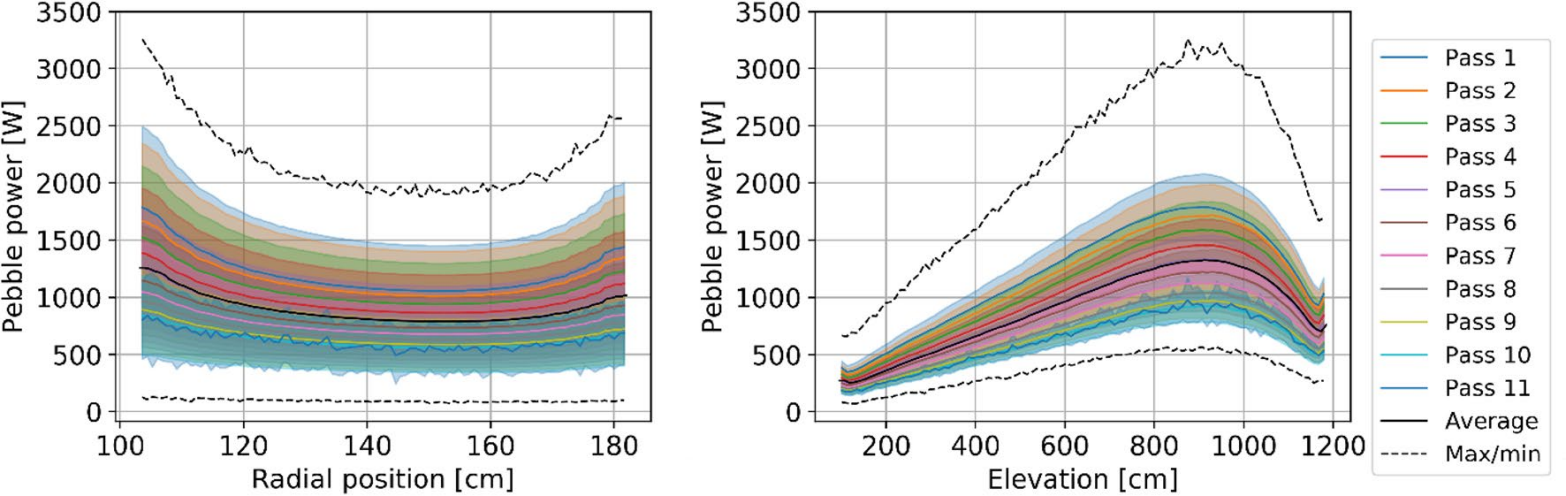
Radial (left) and axial (right) pebble power distribution per pass at equilibrium.

Power production in each pebble is a critical in-core metric as high power production in a zone of the core leads to hot spots, resulting in lower thermal margins for both fuel and coolant temperatures and increased thermal stress on structural materials and reflectors. Figures 16 and 17 illustrate the spatial distribution of power per pebble in the core at equilibrium. The radial and axial profiles show similar shapes to the thermal neutron flux, yielding a roughly constant peaking factor. At every pass, power decreases, in line with what was shown for burnup. On average, at the end of life, a pebble generates half of the power produced during the first pass (Table 5). The first four passes account for half of the total core power (202 MW), pass five to eight for 37% (145 MW), and the last two for 13% (53 MW).

**Table 5:**
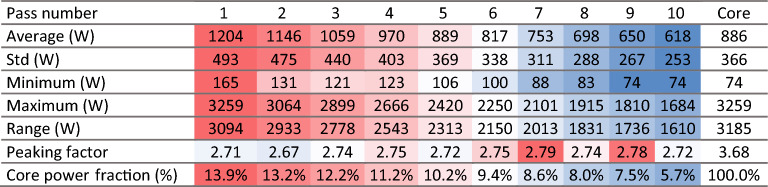
Pebble power statistics as a function of the number of passes.

The colors are mapped from blue (lowest values) to red (highest values).

**Figure 18:**
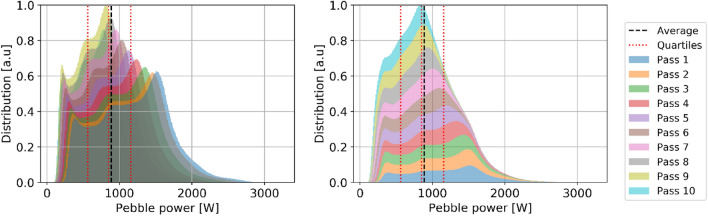
Pebble power statistical distribution per pass at equilibrium, individual pass (left), and cumulative (right), normalized over the maximum count.

The statistical distribution of pebble power for each pass (Fig. 18) shows three peaks representing distinct thermal flux regions, as seen above. It becomes closer to a uniform distribution due, once again, to the random radial re-insertion process.

Finally, it is observed that the peak power per pebble in the core is 3259 W, corresponding to 217 mW per TRISO particle. This information is particularly relevant to assess fuel performance. This work assumes a fixed uniform temperature distribution, but in the future, coupling with a thermal–hydraulic model will be implemented to determine the implication of a detailed pebble-by-pebble power distribution.

#### Data for individual pebbles

**Figure 19:**
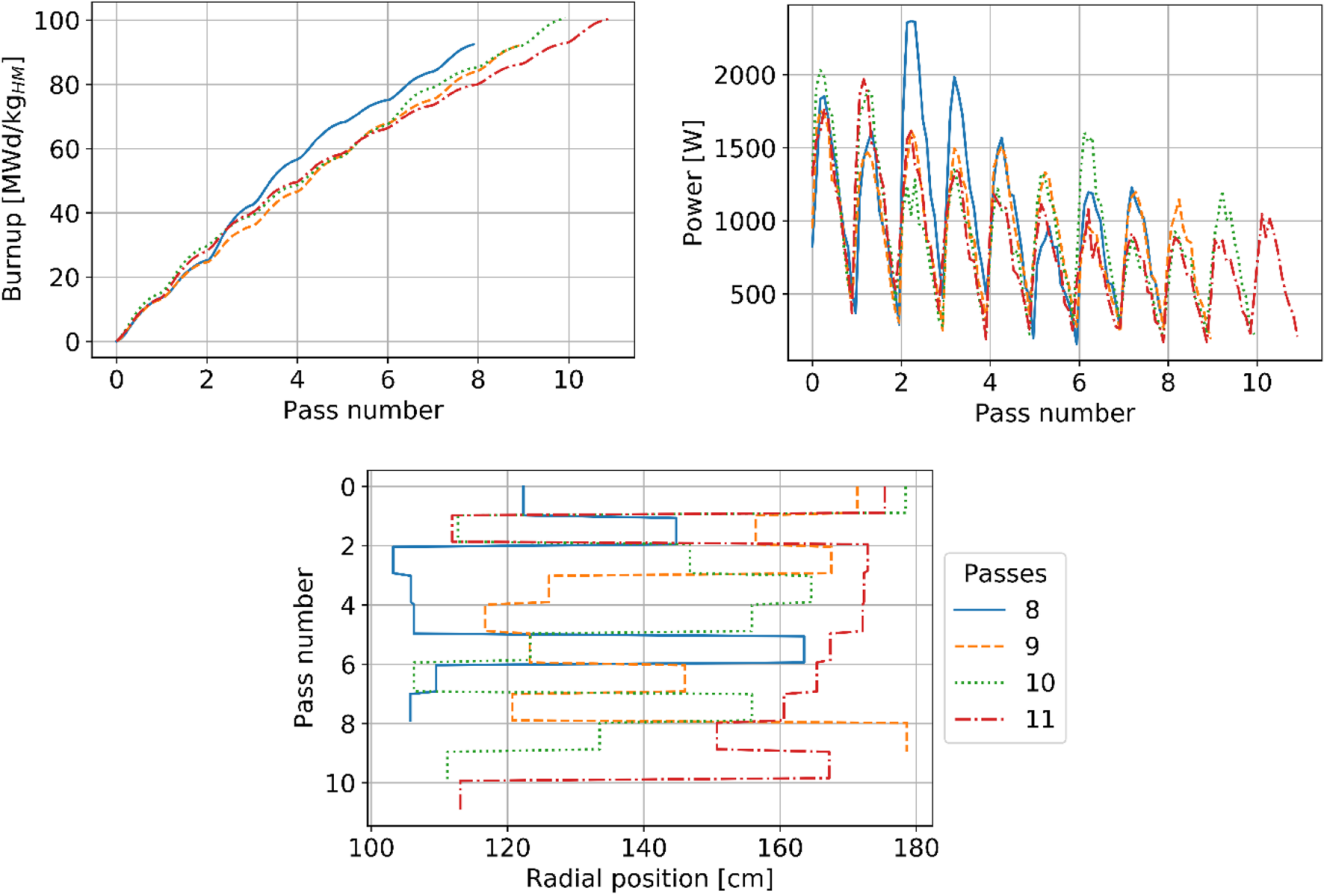
Burnup (top left), power (top right), and radial position (bottom) evolution of selected pebbles being discharged after 8, 9, 10, and 11 passes.

A unique capability of HxF is the possibility to pinpoint the history of every single pebble providing further insights. As an example, data are presented for four pebbles to understand why they were discarded after four different numbers of passes. Their history in terms of burnup, power, and spatial position is shown in Fig. 19. The pebble discharged after eight passes travels mostly close to the inner reflector; therefore, it experiences larger flux/power and accumulates burnup more rapidly. At the opposite extreme, the pebble discharged after 11 travels further away from the reflector and, from pass five on, moves more and more into the lower power region. Notably, although pebbles discarded after 8 or 9 passes accumulated a burnup of about 92 MWd/kg_HM_, the other two reached about 101 MWd/kg_HM_, in line with an extra pass.

### Used fuel data

**Figure 20:**
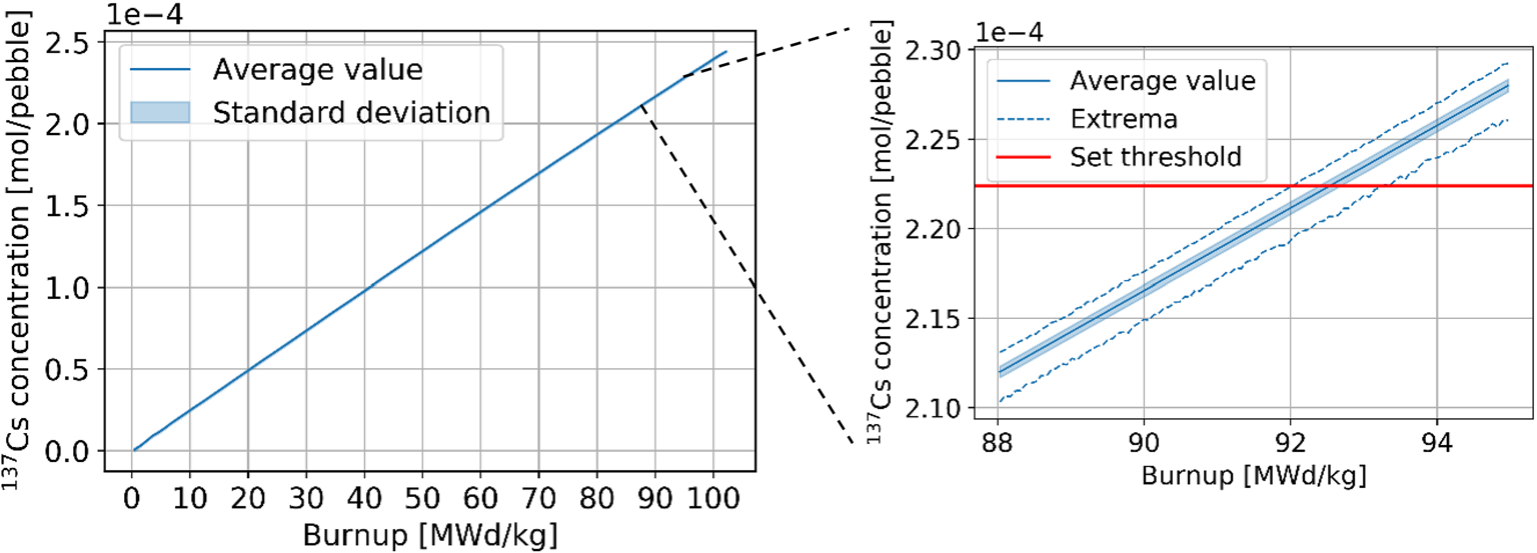
^137^Cs in a pebble as a function of burnup.

In addition to high-fidelity in-core data, HxF can be used to collect data on used fuel. First of all, some considerations can be made on the discharge burnup. As explained in the methodology, ^137^Cs concentration is used as a surrogate for burnup, and pebbles are discharged based on a set threshold. The linear relation between burnup and Cs is confirmed from the discharged pebbles data (Fig. 20). The set threshold of 2.2238 × 10^−4^ mol/pebble corresponds, on average, to a burnup threshold of 92.5 +/− 0.15 MWd/kg_HM_ (ranging from 92.0 to 93.3 MWd/kg_HM_).

**Figure 21:**
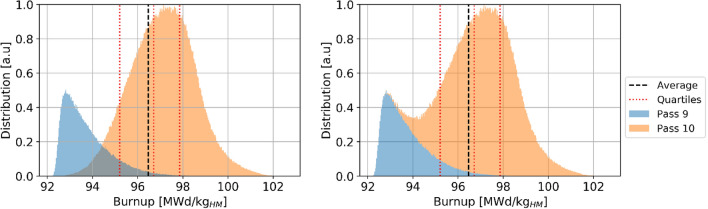
Discarded pebbles burnup statistical distributions per pass, individual pass (left) and cumulative (right).

The ^137^Cs threshold represents the minimum value a pebble must contain in order to be discarded when it is assessed for burnup. Most pebbles are discarded with larger concentrations/burnups, as the assessment occurs only after a full pass. Table 6 summarizes the pebble inventory and burnup information, separated by the number of passes after which the pebbles were discarded. It can be observed that the great majority of pebbles (99.96%) go through the core 9 and 10 times, the average number of passes is 9.8, and the average discarded burnup is 96.5 MWd/kg_HM_, which is 4% higher than the threshold (understanding this shift is important when determining the threshold value). An extremely low number of pebbles (0.03%) goes through the core for 11 passes and typically reach higher burnup levels or are discarded only after 8 and tend to reach lower burnups. In both extreme cases, the obtained burnup ranges are relatively narrow. The statistical distribution of burnup in used pebbles (Fig. 21) shows the threshold cut around 92.5 MWd/kg_HM_ (with the uncertainty discussed above) and two peaks corresponding to discharge after nine or ten passes.

**Table 6:**
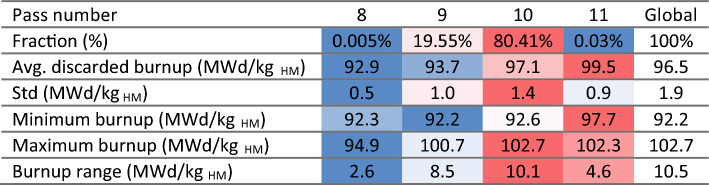
Discarded pebbles inventory and burnup at equilibrium.

The colors are mapped from blue (lowest values) to red (highest values).

**Figure 22:**
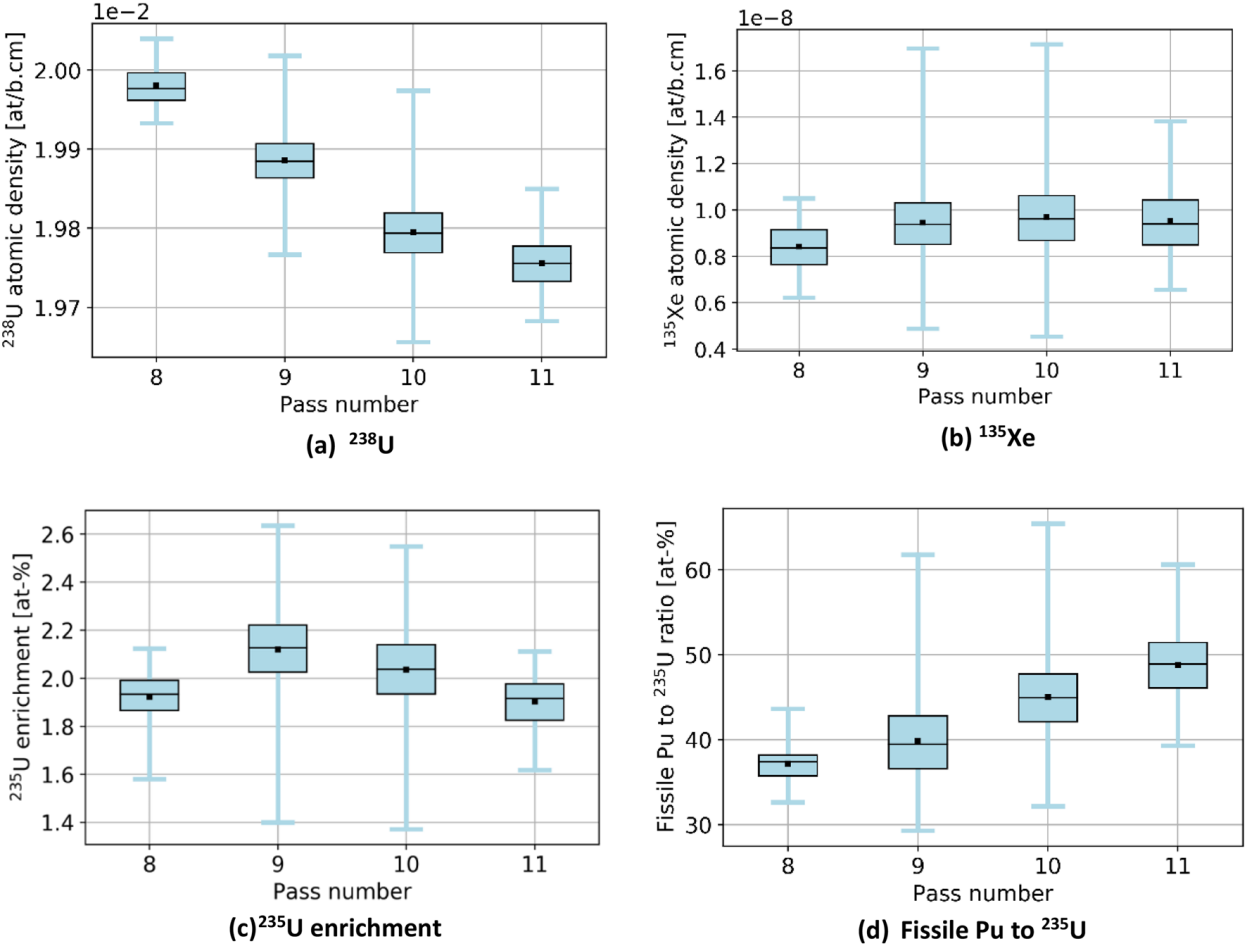
Statistical distributions of isotopic concentrations in discarded pebbles, per pass, for important fuel utilization-related quantities.

**Figure 23:**
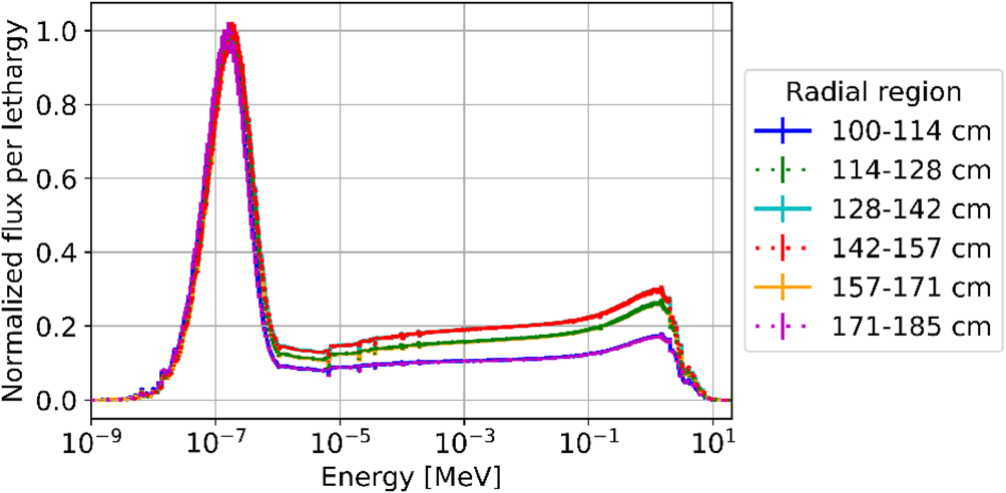
Neutron flux spectrum at equilibrium in six different radial zones.

Information about each individual nuclide can also be obtained. Figure 22 shows a few selected examples (the data are collected at discharge with no decay time). The concentration of ^238^U monotonically decreases with the number of passes as expected, whereas the fissile isotopes of Pu (^239^Pu and ^241^Pu), fission product ^135^Xe, and ^235^U exhibit more complex behaviors. This is due to the diversity of neutron spectra a pebble can experience during its lifetime, depending on the location and, thus, on the trajectories in the core. As previously shown, the pebbles discharged after eight passes are exceptional cases in which the pebbles are mainly located near the reflectors and experience a softer spectrum (Fig. 23). Similarly, a softer spectrum leads to a more efficient consumption of ^235^U and destruction of ^135^Xe.

## Conclusions

A second step towards achieving hyper-fidelity depletion for pebble bed reactors was presented. In this work, pebble motion is represented using an ordered bed and fixed positions through which fuel elements move. Such discrete motion scheme was implemented in Serpent 2 and was combined with its neutron transport and depletion capabilities. Ad-hoc routines were implemented to ensure compatibility with domain decomposition and to handle fresh pebbles insertion and pebble recirculation after each pass based on diverse discharge criteria. The developed discrete motion approach is agnostic to the direction of pebble motion, thus, compatible with any type of PBR. The current simplifications assume no radial motion, uniform axial pebble speed, and uniform core dimensions. However, discrete motion could be further developed to allow for radial shift, to vary pebble speed for modeling phenomena such as wall effects, and to incorporate core regions of different dimensions, such as defueling conic regions. Nevertheless, the authors believe these aspects are better addressed using DEM. Keeping the discrete motion approach simpler provides an alternative to the couple DEM/Serpent that is relatively less demanding in computing time.

A demonstration of the capabilities of HxF with discrete motion was provided using a full-scale HTGR model. More specifically, an approach to equilibrium was performed, and example results were shown both for in-core and discarded pebbles. The data illustrates how HxF provides unique insight into PBR fuel, producing information on statistical distributions rather than average values only, as obtained by traditional methods that rely on spectral zoning for depletion. Knowledge of the distribution, minimum/maximum values, and number of occurrences for parameters such as pebble power and fission product concentration are key data when assessing reactor performance in normal and off-normal conditions and can greatly reinforce confidence in the safety case of PBRs. Furthermore, the data generated does not represent a single equilibrium state but multiple of them (297 in this case), partially addressing the issue that PBRs can assume different configurations even when at equilibrium.

Future work is focusing on further developing HxF for PBRs by improving two aspects. First, DEM will be employed, in place of discrete motion, for a more realistic representation of pebbles' trajectories. In exchange for a higher computational cost, it is expected that DEM will yield more accurate velocity profiles and enable greater flexibility in terms of geometry. This approach avoids relying on assumptions, such as creating lattices of pebbles or predefined flow channels. Second, a thermal–hydraulic solver will be used to derive the temperature distribution in the core instead of assuming a uniform one, as currently done.

## Data Availability

The datasets generated during and/or analyzed during the current study are available from the corresponding author on reasonable request.
